# Seasonal and diurnal patterns of non-structural carbohydrates in source and sink tissues in field maize

**DOI:** 10.1186/s12870-019-2068-4

**Published:** 2019-11-21

**Authors:** Xiao-Gui Liang, Zhen Gao, Li Zhang, Si Shen, Xue Zhao, Yun-Peng Liu, Li-Li Zhou, Matthew J. Paul, Shun-Li Zhou

**Affiliations:** 1 College of Agronomy and Biotechnology, China Agricultural University, Beijing, 100193 China; 2 Current address: Plant Science, Rothamsted Research, Harpenden, Hertfordshire, AL5 2JQ UK; 3 School of Bioengineering, Binzhou University, Binzhou, 256600 Shandong China; 4 Scientific Observation and Experimental Station of Crop High Efficient Use of Water in Wuqiao , The Ministry of Agriculture and Rural Affairs, Wuqiao, 061802 China

**Keywords:** Starch, Non-structural carbohydrates, Photosynthesis, Carbon allocation, Maize, Hybrids, Ontogeny

## Abstract

**Background:**

Carbohydrate partitioning and utilization is a key determinant of growth rate and of yield in plants and crops. There are few studies on crops in field conditions. In *Arabidopsis*, starch accumulation in leaves is a negative indicator of growth rate.

**Results:**

Here, we wished to establish if starch accumulation in leaves could potentially be a marker for growth rate and yield in crops such as maize. We characterized daily patterns of non-structural carbohydrate (NSC) at different growth stages over two seasons for maize hybrids in the field. In 27 commercial hybrids, we found a significant negative relationship between residual starch in leaves and plant growth, but not with final yield and biomass. We then focused on three typical hybrids and established a method for calculation of C turnover in photosynthetic leaves that took into account photosynthesis, leaf area and NSC accumulation. The ratios of stored NSC decreased from approximately 15% to less than 4% with ongoing ontogeny changes from V7 to 28 days after pollination.

**Conclusion:**

The proportion rather than absolute amount of carbon partitioned to starch in leaves at all stages of development related well with yield and biomass accumulation. It is proposed that screening plants at an early vegetative growth stage such as V7 for partitioning into storage may provide a prospective method for maize hybrid selection. Our study provides the basis for further validation as a screening tool for yield.

## Background

Plants coordinate carbohydrate availability between growth and maintenance during daily cycles and over the whole life cycle largely in the form of non-structural carbohydrate (NSC) [1–5]. There are three metabolic fates for immediate photo-assimilate carbon in leaves: a) direct consumption in respiration, b) export for growth mainly as sucrose, and c) storage in leaves in the form of starch and total soluble carbohydrates (TSC), which are transported to various sinks at night [6]. In many species, such as *Arabidopsis*, the major carbohydrate that is transiently stored in leaves is starch, which has near linear rates of synthesis and breakdown during the day-night cycle [7–9]. It was shown that daily starch turnover correlates with the relative growth rate or biomass accumulation in *Arabidopsis* [10–12]. This is potentially a very interesting observation which probably hold in other species including crops and could be used to screen for traits such as crop growth rate and yield.

Maize is a major crop that provides nutrition for humans and livestock and is a source of bioenergy. The forms of carbohydrates that accumulate in leaves of maize are different from those in *Arabidopsis* and other crops such as wheat and soybean [9, 13]. Considerable amounts of both starch and soluble sugar turnover were observed during the day-night cycle in maize leaves [14–16], implying that both would need to be taken into consideration to understand the interrelationship with maize crop growth strategy. It is also noteworthy that NSC turnover may differ within genotypes and developmental stage in certain species [17]. In *Arabidopsis*, the negative correlation between starch accumulation and biomass reflects the larger proportion of newly fixed carbon into sucrose export in faster growing varieties [1, 10, 12].

The quantification of primary photosynthetic carbon assimilation and the ratios of carbon allocation into storage starch and soluble sugars, and transport are not well studied in crops particularly in relevant field conditions. We wanted to establish first the extent to which accumulation of NSC in leaves changed with growth ontogeny and in different hybrids and to relate this to growth, biomass and yield with a long-term goal of using this as a selection tool. For this to be a realistic aim in crop improvement, the development of a final screen would need to use young plants. Using 27 maize hybrids, we first examined the relationship between residual starch in most recently fully expanded leaves and relative growth rate, yield and biomass accumulation. In maize as in *Arabidopsis* a negative correlation between residual starch and relative growth rate was found but not between residual starch and either biomass accumulation or yield. In selected hybrids we then examined in greater depth diurnal patterns of leaf photosynthesis and NSC concentrations and using the data developed a calculation method of NSC turnover. When starch turnover was calculated as a proportion of fixed carbon, good relationships with maturity biomass and yield were found both in young and mature plants. We propose this may provide a basis of a selection marker for yield in maize and potentially other crops.

## Results

### Starch accumulation in 27 maize hybrids

**Fig. 1:**
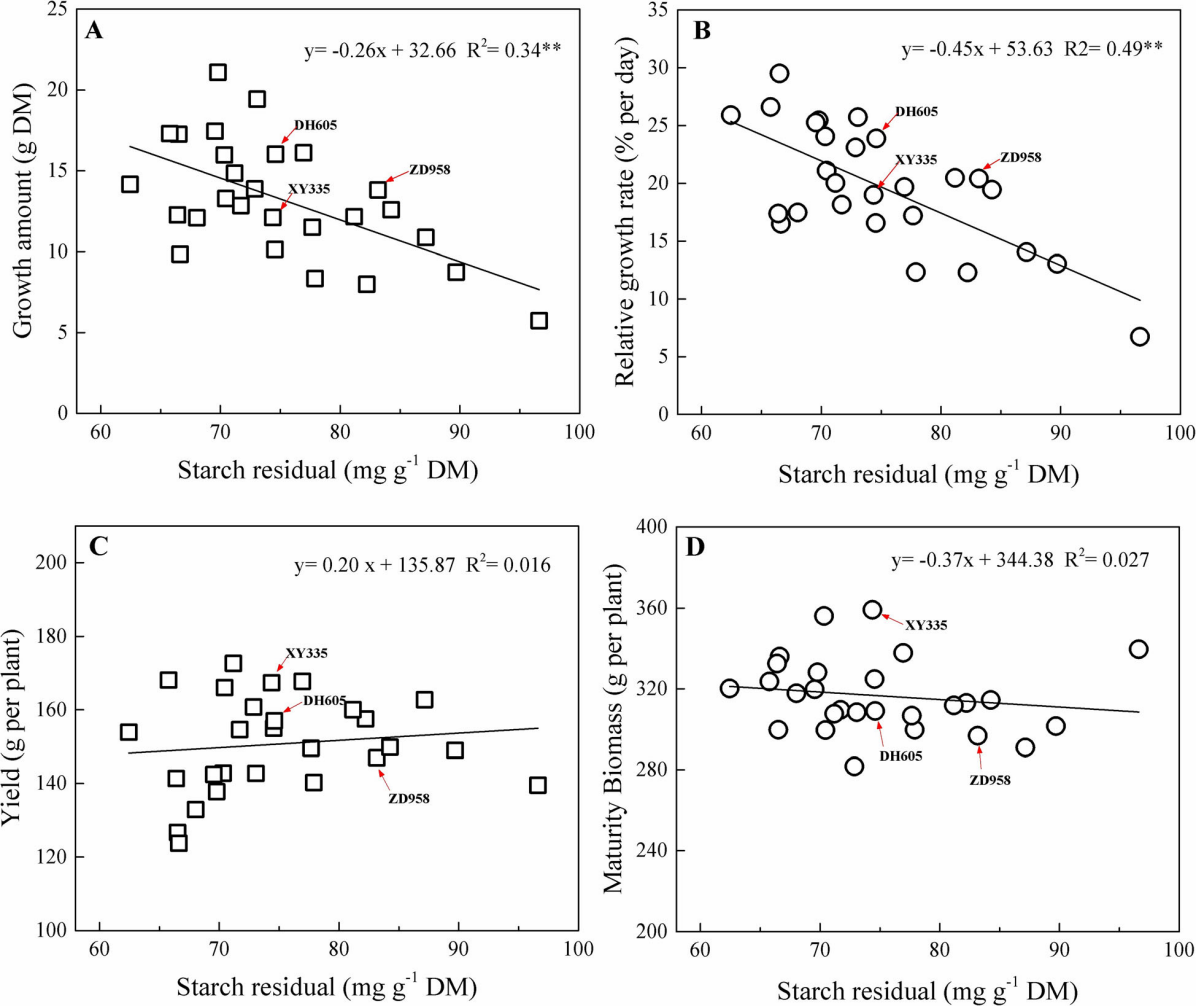
Relationship of residual starch at the 9^th^ leaf expanded stage with the corresponding growth amount (**a**), relative growth rate (**b**), final yield (**c**) and maturity biomass (**d**) for 27 commercial maize hybrids in the 2015 season. Abbreviations: DM, shoot dry matter

As found in *Arabidopsis* [10, 12], significant negative relationships of starch at the end of night (6:00) with growth amounts and relative growth rates were found for the 27 maize hybrids at seedling stage in the 2015 season (Fig. 1). However, there was no significant relationship between residual starch in leaves, and yield and biomass accumulation (Fig. 1). In the next season, we went on to further investigate in greater depth the interrelation between diurnal and seasonal dynamics of carbohydrate accumulation, biomass accumulation and grain yield in three selected maize hybrids. They are now the most widespread in the corn belt of China and were assigned to three different groups based on the cluster analysis of the yield and biomass of the 27 hybrids in 2015 (Additional file 1: Figure S1).

### Plant growth and carbon assimilation

**Fig. 2:**
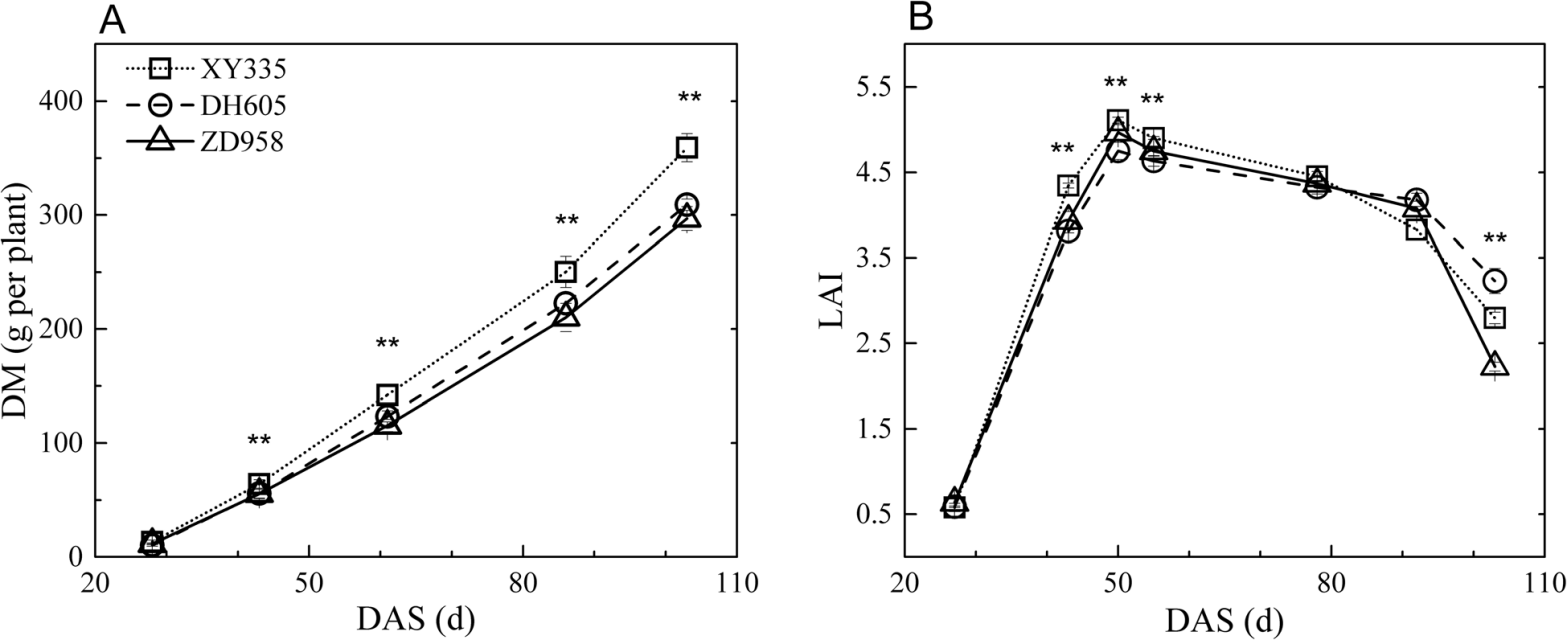
Seasonal changes of total above-ground biomass (DM) (**a**) and leaf area index (LAI) (**b**) in the 2016 season. * and ** indicate significance at *P* ≤ 0.05 and *P* ≤ 0.01, respectively. No significant difference among the points for certain sample time without asterisk marked. Abbreviations: DAS, days after sowing

Total biomass, leaf area and carbon assimilation are proposed to be three fitness proxies [18]. The total above-ground dry biomass (DM) and leaf area index (LAI) in the 2016 season are shown in Fig. 2. The DM values of XY335 were higher than those of the other two hybrids at the later growth stages in both seasons (Fig. 2; Additional file 2: Figure S2). The leaf area quickly reached peak values at approximately the tasselling stage and decreased gradually until later grain filling, and then fell to approximately 55–78% of the previous values in 10 days before maturity. The LAI of XY335 at tasselling time was the highest while that of ZD958 at the maturity stage was the lowest. XY335 was consistently higher yielding, followed by DH605 and ZD958 (Additional file 3: Table S1).

**Fig. 3:**
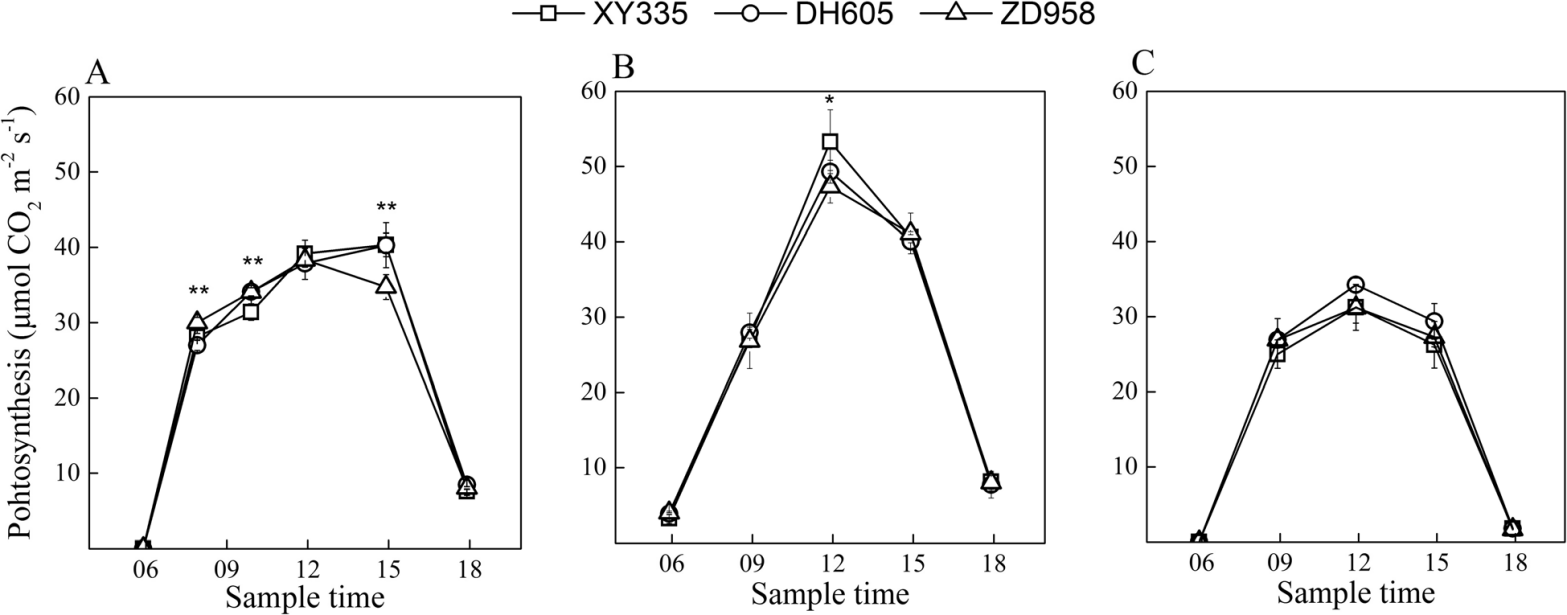
Diurnal changes of net photosynthesis rates in the newly expanded leaves at the periods of 7^th^ leaf expanded (**a**) and 13^th^ leaf expanded (**b**) and the ear leaves at 28 days after pollination (**c**) for maize hybrids. * and ** indicate significant levels at *P* ≤ 0.05 and *P* ≤ 0.01, respectively

The patterns of diurnal photosynthesis of newly expanded maize leaves at the V7 and V13 stages and ear leaf at the 28 days after pollination (DAP) were similar among maize hybrids (Fig. 3). Few notable diurnal differences among modern commercial hybrids were found during the three stages. This result was also confirmed by the sum of net photosynthetic gain per day for each genotype (see below).

### The NSC pool turnover in photosynthetic leaves

**Fig. 4:**
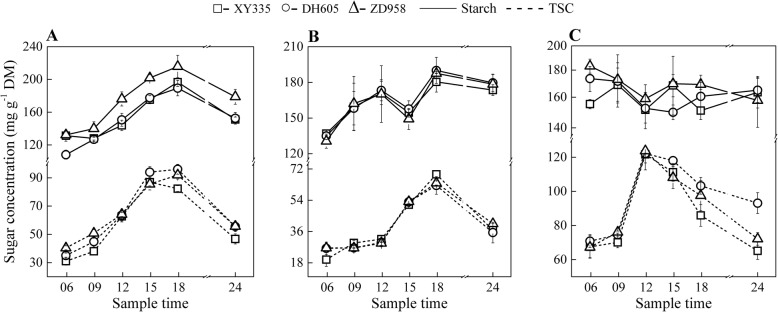
Diurnal changes of starch (solid lines) and total soluble carbohydrates (TSC, short dash lines) in the newly expanded leaves at the periods of 7^th^ leaf expanded (**a**), 13^th^ leaf expanded (**b**) and the ear leaves at 28 days after pollination (**c**). Maize hybrids Xianyu335 (XY335), Denghai605 (DH605) and Zhengdan958 (ZD958) are indicated by hollow square, circle and triangle, respectively

Both starch and TSC accumulated from the beginning of the day (06:00) at the V7 stage (Fig. 4a). The peak of carbohydrate concentrations occurred at the end of the day (18:00) except the TSC of XY335, which reached its maximum concentration approximately at the afternoon (15:00). The starch concentrations of ZD958 were generally higher than that of XY335 and DH605. The starch accumulation pool of ZD958 during the day was 83.4 mg g^− 1^ DM, which was the highest of the three hybrids. The amount of starch accumulation was 56.0% for XY335, 57.8% for DH605 and 61.8% for ZD958 of each total NSC pool at this growth period. The NSC pool stored in the newly expanded leaf during the day was consumed in the following night period. Until midnight (24:00), remobilization of the newly accumulated sugars accounted for approximately 69.6, 55.0 and 54.3% in XY335, DH605 and ZD958, respectively.

The diurnal patterns of starch and TSC concentrations at the V13 stage were similar to that of V7 (Fig. 4b). The values of sugar concentrations were similar among the three hybrids at this rapid growth stage. However, the ratios of starch accumulation to the total NSC pool in the 13^th^ leaf differed greatly among hybrids and were 47.0, 60.4 and 60.4% in XY335, DH605 and ZD958, respectively.

At the period of 28 DAP, the dynamics of the starch concentrations were irregular among hybrids, but rather stable during the day and night, changing in the range of 149.8 to 183.2 mg g^− 1^ DM (Fig. 4c). In contrast, the TSC, which became the obviously predominant form of the transient NSC pool in leaves, accumulated rapidly until noon (12:00) and decreased during the following time (Fig. 4c).

### Temporal levels of the NSC in the main sink and transport organs

The main sink organs were not the same tissue at different growth periods. Younger stems were chosen as the dominant consumer organs at the periods of V7 and V13, and grains were regarded as the main consumer of carbohydrates at 28 DAP. In addition, the main transport organs were sheaths at vegetative stages (V7 and V13) and ear stems at reproductive stage (28 DAP), respectively.

**Fig. 5:**
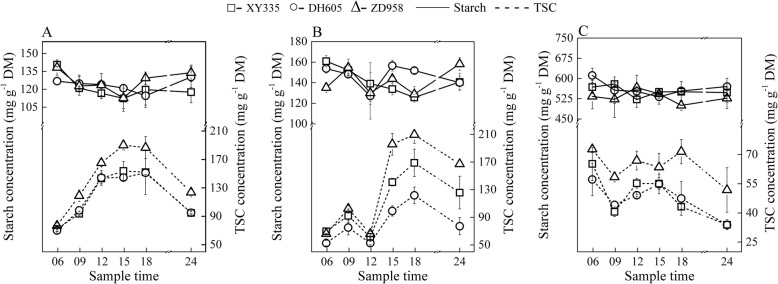
Diurnal changes of starch (solid lines) and total soluble carbohydrates (TSC, short dash lines) in the main sink tissues at the periods of 7^th^ leaf expanded (**a**), 13^th^ leaf expanded (**b**) and 28 days after pollination (**c**). Stem and grain were characterized as sink at vegetative and reproductive stages, respectively. Maize hybrids of Xianyu335 (XY335), Denghai605 (DH605) and Zhengdan958 (ZD958) are indicated by hollow square, circle and triangle, respectively

**Fig. 6:**
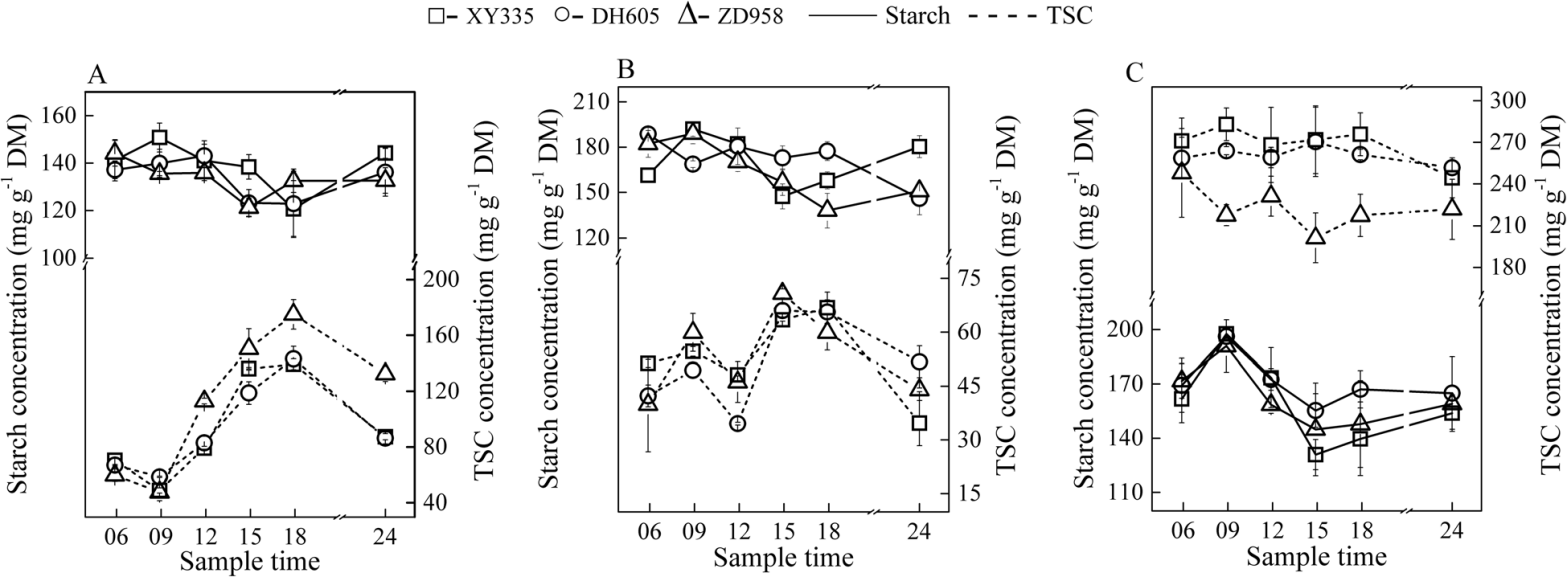
Diurnal changes of starch (solid lines) and total soluble carbohydrates (TSC, short dash lines) in the transport tissues at the periods of 7^th^ leaf expanded (**a**), 13^th^ leaf expanded (**b**) and 28 days after pollination (**c**). Sheath and stem were characterized as transport tissues at vegetative and reproductive stages, respectively. Maize hybrids of Xianyu335 (XY335), Denghai605 (DH605) and Zhengdan958 (ZD958) were indicated by hollow square, circle and triangle, respectively

Diurnal changes in the NSC concentrations in the main sinks (younger stems at V7 and V13, grains at 28 DAP) were rather different from that of source leaves (Fig. 5). Diurnal starch concentrations were relatively stable in both stems and grains, while the diurnal patterns of TSC were different at each sample date. Soluble sugars play the primary role for metabolism in these sinks though the dynamics of the TSC at 28 DAP was relatively constant. The different characteristics of TSC between 28 DAP and V7 and V13 may be mainly the consequence of tissue differences and seasonal change of environment. Interestingly, the TSC in sink organs of ZD958, the lower-yield variety, was generally higher than those of XY335 and DH605 across the three growth stages (Fig. 5). The diurnal patterns of NSC content in transport organs were similar to that of sinks (Fig. 6). Nevertheless, the soluble sugars in transport organs were similar within hybrids at V13 but were generally lower at V7 stage and higher at 28 DAP for ZD958 than for the other two hybrids. Temporal levels of NSC accumulation in other tissues were also presented (Additional file 4: Figure S3) for seasonal comparisons (see discuss below).

### Primary carbon assimilation and allocation

Based on the calculation method developed for maize photosynthetic leaves, the amount of carbon assimilation during the day and the participation of sugars into transport and transient NSC storage (starch and TSC) were determined for each hybrid at each sample date (Table 1). An example of the detailed processes of calculation and conversion can be found in Table 2.

**Table 1:** Diurnal carbon assimilation and allocation at different growth periods

Growth period	Hybrids	*A*_*D*_	*C*_*T*_	*C*_*T*_/*A*_*D*_	*C*_*A*_/*A*_*D*_	Starch/*A*_*D*_	TSC/*A*_*D*_
		μmol C g^−1^ DM	μmol C g^− 1^ DM	%	%	%	%
V7	XY335	29.32	25.43	86.73	13.27	7.43	5.84
	DH605	29.66	24.97	84.20	15.80	9.13	6.66
	ZD958	28.38	23.88	84.14	15.86	9.80	6.07
V13	XY335	25.29	22.20	87.81	12.19	5.73	6.46
	DH605	25.55	22.51	88.10	11.90	7.18	4.72
	ZD958	25.41	22.26	87.62	12.38	7.48	4.90
28 DAP	XY335	14.39	13.92	96.75	3.25	−1.01	4.26
	DH605	15.16	14.51	95.71	4.29	−2.84	7.12
	ZD958	14.81	14.26	96.34	3.66	−3.17	6.83

*A*_*D*_ represents the total net carbon accumulation that was calculated by diurnal photosynthesis values, *C*_*T*_ represents the transported sugars and was the result of *A*_*D*_ minus *C*_*A*_, and *C*_*A*_ represents carbon accumulation in certain maize leaves, which was approximately equal to the non-structural carbohydrates (NSC) concentration. Hence, *C*_*A*_ was calculated by the sum value of starch and total soluble carbohydrates (TSC). The ratios of transport, accumulation, starch and total soluble carbohydrates participation were summarized. Abbreviations: V7, the 7^th^ leaf expanded stage; V13, the 13^th^ leaf expanded stage; 28 DAP, 28 days after pollination

**Table 2:**
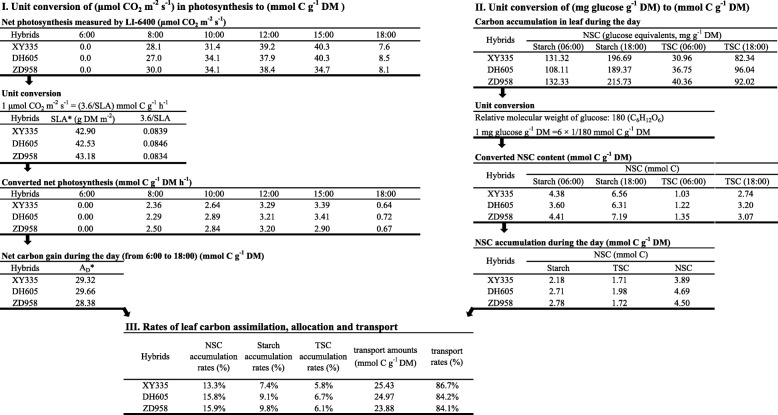
Process of the calculation method for daily carbon turnover in maize photosynthetic leaves

In the 1^st^ and 2^nd^ steps, the daily photosynthesis and sugar accumulation were transformed into unified unit (mmol C g^−1^ DM). Then the ratios were quantified in the 3^rd^ step. XY335, DH605 and ZD958 represent the three hybrids used in this study. Abbreviations: DM, dry matter; SLA, specific leaf area; *A*_*D*_, sum of daily photosynthesis, i.e. the net carbon assimilation; NSC, non-structural carbohydrates; TSC, total soluble carbohydrates

Total amounts of C assimilation (*A*_*D*_) were similar among hybrids for a given period, showing little discrepancy in the capacity of photosynthesis of a single leaf among modern maize hybrids. However, the allocation of carbon into storage and transport differed in leaves. The ratios of starch turnover to *A*_*D*_ (*Starch/A*_*D*_) for XY335 were constantly lower than those of DH605 and ZD958 at the three sample stages, while the ratios of TSC (*TSC/A*_*D*_) were irregular among hybrids. During development, the ratios of starch accumulation were highest at V7, and then tended to decrease and eventually disappear at 28 DAP. However, the TSC ratios were nearly constant throughout the growth periods, ranging from 4.26 to 7.12% for different hybrids (Table 1). In total, the ratios of daily NSC accumulation (*C*_*A*_/*A*_*D*_) in source leaves decreased from approximately 15% to less than 4%, indicating that the transport ratio of immediately fixed carbon gradually increased with the progression of ontogeny.

## Discussion

### Changeable NSC pool in leaves of field maize

Carbohydrate allocation from leaves to the harvested sinks is an important determinant of yield in maize and other crops [19–22]. There are very few studies of carbohydrate allocation in crops growing in the field. Here we performed initially an analysis of 27 commercially available hybrids and then a more detailed analysis with three of these. Observations in *Arabidopsis* have shown a robust correlation between leaf starch turnover and growth rate or biomass accumulation [10–12] which could translate into yield in a crop.

The biological differences between maize and *Arabidopsis* are large, and their daily and seasonal models of sugar metabolism could be different. Although there are similar patterns of starch and TSC concentrations at the V7 stage between the maize leaf (Fig. 4a) and *Arabidopsis* [8, 11], the ratios of starch and TSC in diurnal NSC pools changed along with the transition process from vegetative to reproductive growth periods, which has not been previously reported. At 28 DAP, relatively stable daily starch concentrations were found in maize leaves, while an earlier peak of TSC accumulation was observed at 12:00 rather than that at 18:00 during vegetative stage (V7 and V13) (Fig. 4c). The underlying reasons for the changed carbon accumulation during the season still need further study. Fernandez et al. [23] reported a similar phenomenon that foliar starch turnover was accelerated by day length and low light in *Arabidopsis*. The temperature differences within sample dates (Additional file 7: Figure S4) may also impact carbohydrate turnover. So far, we know little about how maize leaf carbohydrates adjust to diurnal temperature differences. Lower daytime temperature will decrease growth more than photosynthesis, which may increase the proportion of photosynthate allocated to storage carbon [24, 25]. Furthermore, a maize seedling experiment by Sunoj et al. [26] showed few changes in photosynthetic rate and decreased starch concentrations in maize leaves under the variable diurnal mean temperature from 30 to 35 °C. Most likely, differences of starch accumulation versus NSC are mainly due to changed growth ontogeny with some influence of temperature. During the grain filling stage of maize, the fast-growing kernels require large amounts of carbohydrates, which consume sugars that would otherwise accumulate as starch or TSC in leaves.

Starch concentrations in leaves were generally the highest at the early growth period (Fig. 4), in contrast to that of stems and sheaths where starch accumulation increased with ongoing ontogeny (Fig. 5, Fig. 6; Additional file 4: Figure S3). We speculate that these phenomena are correlated with the long-term adaptation and adjustment of the plant itself to environmental changes during growth processes.

### Differences in carbohydrates in sink tissues among hybrids

XY335 and ZD958 are the two most typical and popular commercial maize hybrids in Northern China. In addition, both hybrids possess the traits of good abiotic resistance and high yield potential. In our field study without any pronounced stress conditions, visual differences in carbohydrates were mainly observed in the sink tissues. XY335 accumulated higher above ground biomass than ZD958 (Fig. 2), but the TSC concentration in the sink organs was consistently lower in XY335 than ZD958 (Fig. 5). We tried to explain the differences in carbohydrates in sink tissues by correlating the sugar levels in sinks with the grain weight and yield but failed. It is proposed that although the starch concentrations in sink tissues were similar among hybrids, the sink capacities, i.e., kernel number and grain weight were different. Fast-growing varieties often have higher biomass increase and stronger sink capacity, which may lead to competition for supplied sugars, and hence reduced soluble carbohydrate concentrations [1]. The metabolic mechanism, in terms of source-sink relationship, for explaining the dynamic change of soluble carbohydrates requires further exploration.

### Calculation of carbon turnover for maize photosynthetic leaves

Based on the C-balance model [25, 27] and the approach proposed by Kalt-Torres et al. [28], we created a calculation method of carbon turnover for maize photosynthetic leaves. By measuring diurnal photosynthesis from representative leaf positions and sugar dynamics from the whole leaf, we calculated the daily carbon assimilation and sugar accumulation and in-turn the partitioning for single leaves. Though additional error may be introduced because of the differences along both axes of a leaf, increasing the number of measurement replicates would reduce any error. In our study, the data showed that the transport ratios of carbon mass from maize photosynthetic leaves were more than 80% throughout the growth period (Table 1), confirming previous work. The range of 84.1–86.6% at V7 stage for the three selected hybrids is consistent with previously proposed ratio of 85% carbon export for young maize leaves in the juvenile period by Kalt-Torres et al. [28]. The export ratios increased with the progression of ontogeny, which is explained mainly as a result of increased sink demand. From the vegetative stage to reproductive stage, the transition from sink-growth-limitation to source-produce-limitation occurred. Synchronously with the phase transition, the transport ratios changed from approximately 85% to more than 95% (Table 1). This observation to our knowledge has not been reported before since most research related to carbohydrate coordination with growth was conducted in model plants or crops at seedling stages. Moreover, the calculation method we developed could also be used to include nocturnal carbon costs and transport when respiration is measured during the night. Diurnal “accumulated carbon” for maize plants, which is similar to the term “accumulated temperature”, could be determined by the measurement of total CO_2_ exchange of whole maize plants. The method implemented here may provide a valuable input into crop growth models and how carbon partitioning relates to growth parameters and yield with potential as a selection tool in the future.

### Starch metabolism is tightly correlated with maize growth and yield

**Fig. 7:**
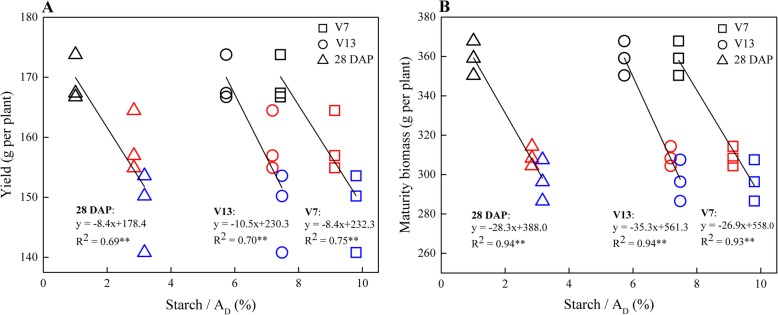
Relationships between final yield (**a**) and biomass (**b**) and the ratios of starch to daily carbon accumulation in leaves at different growth stages. Hollow square, circle and triangle represent for the stages of 7^th^ leaf expanded (V7), 13^th^ leaf expanded (V13), and 28 days after pollination (DAP), respectively. Black, red and blue points represent for XY335, DH605 and ZD958, respectively

Primary carbon partitioning in photosynthetic leaves provides a basis of plant growth and development in *Arabidopsis* [1]. One of our aims was to explore the correlation between starch metabolism and growth and yield, which may provide insights into the regulatory networks relate to physiological, biochemical and metabolic processes for crops such as maize. It could also enrich the valuation indicators and assist selection of varieties. Among hybrids, the ratios of starch to A_D_ (*Starch*/*A*_*D*_) for XY335 were constantly the lowest and those of ZD958 were highest throughout the growth periods (Table 1). We managed to correlate the absolute ratios of starch turnover to daily net carbon fixation with final yield and maturity biomass (Fig. 7). Similar relationships between starch and biomass have been reported in *Arabidopsis* based on the absolute amounts of starch rather than the proportions of starch [10, 12]. Therefore, combining the investigation here and those of the 27-hybrids, we propose that genotypes that reserve less starch in leaves could have higher growth rate. When starch turnover was calculated as a proportion of diurnal carbon assimilation, the relationship becomes negatively related to final yield and biomass itself. This trait for maize has not been reported elsewhere and could be a prospective index for hybrid selection at very early growth stages since the negative relationship is consistent throughout development in the three hybrids (Fig. 7). Nevertheless, it should be pointed out that this result was based on growth conditions in the North China Plain with three representative hybrids. A wider number of sites and genotypes for further validation are required before this can be rolled out in practice.

In maize breeding, a large number of genotypes (mostly hybrids) need to be tested in several environments in test-trials each year, which causes a large economic cost. With this possible selection at early stage such as V7, seedling experiments could be conducted in greenhouse or phytotron before field trials. A microplate-based assay with robotized system allows precise and rapid measurement of starch levels in 400 samples per day [8, 12]. This would make it possible to identify and focus on varieties with higher yield potential in field trials and in-turn save both expense and time. Moreover, it would be beneficial to test commercial inbred lines by our method, since their grain yield is essential for the hybrid-seed production.

## Conclusions

Under field conditions in our study, residual starch in the morning in most recently fully expanded leaves related well with relative growth rate but not with final yield in 27 commercial maize hybrids.

In depth characterization of diurnal turnover of starch, total soluble carbohydrates and photosynthesis was characterized in three selected hybrids and a quantitative model was developed to evaluate the proportion of starch and total soluble carbohydrates to diurnal photo-assimilate accumulation. Using this model a negative relationship was found between the ratios of starch turnover and yield across development stages for the three representative hybrids. This might provide a prospective selection marker for maize which could be developed more widely after further validation.

## Methods

### Study site, maize hybrids and sampling

In the 2015 and 2016 seasons, field experiments were conducted at the Wuqiao Experimental Station of China Agricultural University, located in the Hei-Long-Gang Valley in the North China Plain (116° 36′ 42″ E, 37° 41′ 8″ N). The soil conditions, fertilizer application and field management are presented in Additional file 5: Table S2.

Previous findings on *Arabidopsis* accessions reported a negative interrelationship between starch accumulation and growth. Hence, we first examined whether similar relationships existed between plant growth and starch content at the end of the night. Twenty-seven commercially released maize hybrids (Additional file 1: Figure S1), which are currently widespread in the North China Plain, were bought from the local markets and planted in the field trial according to the completely randomized block design in three replications. Final plant density was 67,500 plants ha^− 1^. Each sub-plot consisted of six rows with 9 m^2^. Samples were taken at seedling stage in the 2015 season. Starch concentrations of expanded leaves and shoot dry matter (DM) were measured at dawn (06:00) and at dusk (18:00) on the 1^st^ and 3^rd^ day of full expansion of the ninth leaf. Starch concentrations were related to growth amounts and relative growth rates.

For in-depth analysis, we compared three maize genotypes, Xianyu335 (XY335), Denghai605 (DH605) and Zhengdan958 (ZD958) in the 2016 season (Additional file 6: Table S3). These hybrids were chosen because they are three of the most dominant commercial maize hybrids in the corn belt of China at present and according to the result of cluster analysis of the 27 hybrids for yield and biomass, they belong to three different groups (Additional file 1: Figure S1).

In the 2016 season, sampling and measurement was at the stages of 7^th^ leaf fully expanded (V7), 13^th^ leaf fully expanded (V13) and 28 days after pollination (DAP), representing the vegetative stage, transitional stage and middle reproductive stage, respectively. Plants were sampled six times within 24 h, approximately every 3 h in daytime (06:00, sunrise; 09:00, morning; 12:00, midday; 15:00, afternoon) and twice at night (18:00, sunset; 24:00, midnight) during consistently sunny days. For each time point, the above-ground parts of pre-marked typical plants with replicates were cut and separated within 30 min into the upper expanded leaf (V7 and V13) or ear leaf (28 DAP), older expanded leaves, unexpanded leaves (V7 and V13), stems and grains (28 DAP). Then, the bagged samples were rapidly heated to denature enzymes and dried at 70 °C for 48 h to constant weight as previously reported [17, 29]. Seasonal changes in total DM and leaf area index (LAI) were also determined in the 2016 season for the three hybrids.

### Photosynthesis and NSC measurements

In the 2016 season, net photosynthesis rates (*A*) of newly fully expanded leaves or ear leaves after pollination were measured by an LI-6400 (LI-COR, USA) with a standard (2*3 cm) leaf cuvette for the three hybrids at the time points of 6:00, 9:00, 12:00, 15:00 and 18:00 with the sets of flow (400 umol s^-^^1^), Ref CO_2_ (400 umol mol^− 1^), Tblock: 20 °C and PPFD (artificial light intensity used according to the environment). Clear and cloudless days were chosen for each stage and the specific light intensity and temperature for each diurnal measurement was also presented (Additional file 7: Figure S4). Measurements for the middle part of leaves were taken in less than 1 h at each time point. Average rates were determined for four replicates from separate plants of each hybrid. Starch and total soluble carbohydrates (TSC) were determined by anthrone-sulfuric acid method [30–32] with some modification. The dried tissues were ground to a fine powder and weighed to approximately 0.1 mg; then the samples were boiled three times in distilled water to extract total soluble sugar. Each supernatant was collected and brought up to a constant volume. Starch in the residue was digested with hydrochloric acid in boiling water, neutralized with sodium hydroxide and then diluted to 50 ml. TSC and starch were measured with anthrone-sulfuric acid using spectrophotometer (TU-1901, China) at wavelength of 625 nm [33].

### Calculation method of carbon transportation and storage

A method to estimate carbon export for certain maize leaves has been proposed by Kalt-Torres et al. [28], who calculated the transport rate by carbon assimilation and the variation in leaf disk dry weight during a certain interval. However, they did not assess the total fixed C and the allocation between starch and soluble sugar. It has been considered difficult to measure accurately dry weight in field conditions with small leaf disks. Based on a C-balance model, which was established by Pilkington et al. [25] and Sulpice et al. [27] and was used to estimate the rate of growth during the day and night for *Arabidopsis*, we proposed a method to calculate carbon turnover for maize leaves.

The total amount of C assimilation (*A*_*D*_) during the day can be estimated as the sum area of net photosynthesis [*A*_*D*_ = ∑ (*A* × Hours)]. The assimilation export rate (*C*_*E*_) can be estimated as *A*_*D*_ minus the C accumulated in leaves (*C*_*E*_ = *A*_*D*_-*C*_*A*_). *C*_*A*_ represents the sum of daily variation of C accumulated in starch, soluble sugar, amino acids, organic acids. We did not consider amino acids and organic acids [27]. Hence, *C*_*A*_ is the sum of starch and total soluble sugars [27].

One of the critical feasibility factors in this method was the unit conversion from μmol CO_2_ m^− 2^ s^− 1^ into μmol CO_2_ g^− 1^ s^− 1^ of net photosynthesis by the ratio of specific leaf area (SLA), which may be more accurate and representative than small sample disks. Hence, area and relevant dry weight of the leaf measured for photosynthesis were determined for each hybrid. The other essential factor was the constant content of molecular C from CO_2_ to (CH_2_O) irrespective of the existence of different forms of sugars.

### Yield and yield components

At maturity, maize ears were hand harvested to determine yield (14% water content) in each plot with 6 square meters (2 rows × 5 m length). All the harvested plants were surrounded by 2 border rows. Yield components including ear length, ear rows and kernels per row were measured based on 10 selected representative maize ears. After drying kernels in 70 °C to constant weight, 500-grain weight was determined.

### Statistical analysis

Data sets were standardized by using functions of Microsoft Excel 2010. SPSS 20 was used for analyses of variance, and Origin 8.0 was used in mapping and curve fitting of figures.

## Supplementary information

**Additional file 1:** **Figure S1.** Cluster analysis using ward linkage by yield and biomass at maturity stage for the 27 commercial hybrids.

**Additional file 2:** **Figure S2.** Seasonal changes of the above ground biomass in the 2015 season (*n* = 3). Abbreviations: DM, dry matter; DAS, days after sowing.

**Additional file 3:** **Table S1.** Yield and yield components in the 2015 and 2016 seasons.

**Additional file 4:** **Figure S3.** Diurnal changes of starch (solid line) and total soluble carbohydrates (TSC, short dash line) concentrations in the rest of the plants at different growth periods. (A) Older expanded leaves but the 7^th^ leaf at 7^th^ leaf stage, (B) Unexpanded leaves at 7^th^ leaf stage, (C) Older expanded leaves but the 13^th^ leaf at 13^th^ leaf stage, (D) Unexpanded leaves at 13^th^ leaf stage, (E) Whole leaves but the ear leaves at 28 days after pollination, and (F) Whole stems but ear stems at 28 days after pollination.

**Additional file 5:** **Table S2.** Details of soil conditions in the upper 20 cm before sowing, fertilizer application and field management.

**Additional file 6:** **Table S3.** Details of the three maize hybrids used in the 2016 season.

**Additional file 7:** **Figure S4.** Diurnal changes of light intensity and temperature during the sampling days. Lines shown were fitted by ß-spline. Abbreviations: V7, the 7^th^ leaf expanded stage; V13, the 13^th^ leaf expanded stage; 28 DAP, 28 days after pollination.

## Data Availability

Additional data are available in Additional files 1, 2, 3, 4, 5, 6 and 7.

## Abbreviations

*A*: Net photosynthesis rate
*A*_*D*_: Daily amount of photo-assimilate
C: Carbon
*C*_*A*_: Daily C accumulation
*C*_*E*_: Daily C export
DAP: Days after pollination
DAS: Days after sowing
DM: Dry matter
LAI: Leaf area index
NSC: Non-structural carbohydrates
SLA: Specific leaf area
TSC: Total soluble carbohydrates
V13: 13^th^ leaf fully expanded
V7: 7^th^ leaf fully expanded
