# Supplementary figures and images for: Seasonal and diurnal patterns of non-structural carbohydrates in source and sink tissues in field maize
Source: BMC Plant Biol. 2019 Nov 21;19:508. doi: 10.1186/s12870-019-2068-4 (PMC6868840; doi:10.1186/s12870-019-2068-4)

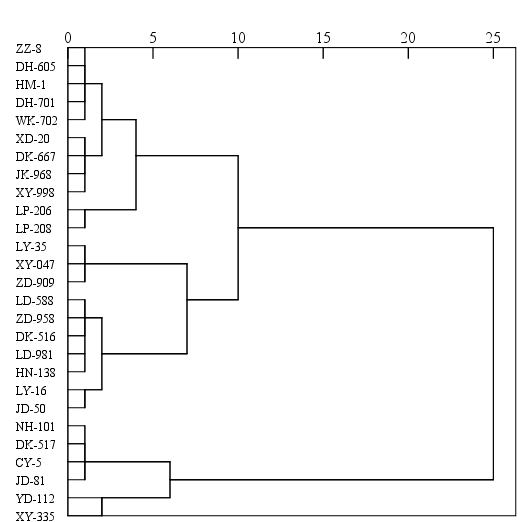

Supplement: Supplementary file 1 — Additional file 1: Figure S1. Cluster analysis using ward linkage by yield and biomass at maturity stage for the 27 commercial hybrids. [file 12870_2019_2068_MOESM1_ESM.tif]
